# Role of artificial intelligence in determining nutritional risk factors among post-periodontal surgical patients. A scoping review

**DOI:** 10.3389/froh.2026.1748346

**Published:** 2026-02-05

**Authors:** Sudhir Rama Varma, Prabhu Natarajan, Jayaraj Kodangattil Narayanan, Ruba Odeh

**Affiliations:** 1 Department of Clinical Sciences, Ajman University, Ajman, United Arab Emirates; 2 Center for Medical and Bio-Allied Health Sciences Research, Ajman University, Ajman, United Arab Emirates; 3 Department of Basic Sciences, Ajman University, Ajman, United Arab Emirates

**Keywords:** artificial intelligence (AI), nutrition, nutritional risk factors, post-periodontal surgery, nutritional risk

## Abstract

**Introduction:**

This scoping review examines recent peer-reviewed literature (2019–2025) on the role of artificial intelligence (AI) in managing nutrition care for post-periodontal surgical patients, and identifies key risk factors influencing nutritional outcomes after periodontal surgery. AI modalities considered include machine learning, expert systems, clinical decision support, and predictive analytics.

**Methodology:**

A systematic search of databases (e.g., PubMed, Scopus) identified studies on AI applications in periodontology, nutrition, or wound healing. The inclusion criteria were English-language, peer-reviewed publications from 2019 onwards that focused on AI in periodontal care or nutritional management, and studies addressing risk factors (such as age, comorbidities, dietary compliance, oral function, socioeconomic status, etc.) that affect post-surgical nutrition or healing. Data were charted on study characteristics, AI type, nutritional outcomes, and reported risk factors. 28 publications were included (10 original studies, eight reviews, five clinical reports, five conceptual papers). AI has been used in periodontal care for diagnostics, prognostics, and decision support.

**Results:**

Machine learning models can predict healing and nutritional risks by analyzing patient data, with key risk factors including age, comorbidities such as diabetes, poor nutrition, low dietary compliance, oral function, and socioeconomic status. Older, chewing-impaired patients have lower nutrient intake and a higher risk of malnutrition. Poor pre-surgery nutrition delays healing. AI models forecast outcomes, identifying baseline pocket depth and antibiotic use as strong predictors. Emerging AI tools in periodontology can enhance nutrition management through early risk detection and personalized diets.

**Conclusion:**

Factors like age, health, oral function, and socioeconomic status affect recovery. Using AI risk assessments with nutritional plans may improve healing. More research is needed to realize AI's full potential. While direct studies are limited, emerging evidence indicates strong potential for personalized, AI-supported nutritional care.

## 1 Introduction

Periodontal surgical procedures, such as flap surgeries, grafts, and regenerative interventions, are commonly used in the treatment of advanced periodontitis and other gum diseases. The post-surgical phase is crucial for the healing of the periodontium (the supporting structures of the teeth), and proper nutrition plays a pivotal role in tissue repair and immune function (1, 2). Malnutrition or suboptimal nutrient intake can impair wound healing, immune response, and recovery. In contrast, an adequate intake of protein, vitamins (A, C, D, etc.), and minerals (such as zinc and calcium) is associated with improved outcomes in periodontal healing. Clinical studies have shown that deficiencies in key nutrients (e.g., vitamin D or protein) correlate with delayed post-surgical periodontal healing. Conversely, nutritional supplements (such as vitamin C, vitamin D, or B-complex) have been investigated for their potential to enhance periodontal wound healing, although the evidence is still evolving (3, 4). Thus, optimal nutrition care management is considered a supportive therapy following periodontal surgery, aiming to promote tissue regeneration, reduce the risk of infection, and improve patient outcomes.

Many patients are recovering from periodontal surgery (2, 5, 6). Face nutritional challenges due to factors like reduced chewing ability from pain, swelling, or sutures, leading to inadequate intake and poor healing. Age-related issues such as oral frailty and slower healing increase risk, especially in older adults who may eat a limited, softer diet. Systemic conditions like diabetes and other comorbidities can impair healing and require special nutritional management. Baseline poor nutrition also raises complications, as it may hinder healing and increase recovery time. Preoperative nutrition optimization or postoperative supplements can improve outcomes. Patient behavior and compliance with dietary recommendations are key factors in achieving successful treatment outcomes. After periodontal surgery, patients are advised to follow a soft or modified diet and consume a balanced diet to support healing. Failing to follow these guidelines, such as eating hard foods too soon or lacking enough protein, vitamins, and fluids, can lead to complications (7–9)—can traumatize healing tissues or cause deficiencies, leading to issues like wound dehiscence or infection. Food preferences, cultural habits, smoking, and alcohol use also affect adherence. Smoking particularly impairs healing, reduces appetite, and compromises nutrient absorption, thereby complicating outcomes. Socioeconomic factors further influence nutrition; low income and limited access to healthy foods often prevent following post-surgery diets rich in protein, produce, or supplements. A recent study links social disadvantages—like low income and food insecurity—to increased periodontitis risk. Both periodontitis and chronic diseases share risk factors such as smoking, poor diet, and socioeconomic challenges, meaning disadvantaged patients with severe gum disease may also find it hard to maintain optimal healing diets (2, 7, 8).

### 1.1 AI and possibility of a customized approach

Addressing these multifactorial risks requires a comprehensive, personalized approach to post-operative nutrition care—one that accounts for the patient's age, health conditions, oral functional capacity, and social context. In this regard, Artificial Intelligence (AI) (9, 10) has emerged as a promising tool to enhance clinical decision-making and personalized patient management in medicine and dentistry. AI refers to computational techniques (including machine learning, deep learning, neural networks, and expert systems) that analyze complex datasets and assist with tasks such as prediction, classification, and pattern recognition. In the context of periodontal care, AI applications have experienced rapid expansion over the last few years. For example, AI-driven image analysis can detect periodontal disease on radiographs with high accuracy, and machine learning models have been developed to predict periodontal treatment outcomes and disease progression using patient data. Such models can process a multitude of variables—from clinical measurements (such as probing depths and attachment levels) to patient characteristics (e.g., age, smoking status, and medical history)—to stratify patients by risk and likely outcomes. This capability aligns with the principles of precision medicine by providing *personalized risk assessments and treatment plans tailored to* each patient. In periodontal maintenance, a scoping review noted that by analyzing combinations of genetic profiles, clinical records, and lifestyle factors, AI can offer personalized care strategies and predict disease susceptibility. AI systems thus have potential not only in diagnosing disease but also in clinical decision support, guiding interventions such as when to perform surgery or how to tailor postoperative care (11, 12).

### 1.2 Role of AI in nutritional approach

AI's role in nutrition care for post-periodontal surgery is a new, interdisciplinary area. AI, mainly used in nutrition for tracking intake, analyzing nutrients, and screening for malnutrition, offers potential in dental aftercare. For example, machine learning applied to health records can flag at-risk patients—such as those with poor healing or nutritional deficiencies—and support early intervention. An AI decision support system could assess a patient's data—such as age, BMI, and health conditions—to generate a “nutritional risk score,” alerting clinicians to high-risk cases that require nutritional plans. It could also personalize dietary advice, predicting optimal protein intake or recommending supplements to improve healing (12–14).

Few studies focus on AI in post-periodontal-surgery nutrition, but insights can be drawn from related research. AI models predict periodontal outcomes; a 2025 study found that baseline pocket depth was a key factor, with additional factors such as tooth type and antibiotic use also influencing healing. While not nutrition-specific, this illustrates AI's ability to identify patient factors that affect healing, which is useful for nutritional analysis. AI also detects malnutrition risk by analyzing multidimensional data. Though its role in nutrition is emerging, AI shows promise for dietary assessment and risk detection. By combining dental and nutritional data, AI can predict healing complications by recognizing complex patterns, such as age over 70, low Vitamin D levels, and uncontrolled diabetes, enabling early intervention.

In summary, the convergence of AI, periodontology, and nutrition presents a novel approach to enhancing healing in patients undergoing periodontal surgery. With the rapid growth of AI research and the importance of nutrition in wound healing, a scoping review is needed to map current knowledge, identify gaps, and suggest how AI can help manage nutritional risks. This review aims to summarize AI applications in periodontal treatment (2), list risk factors, especially nutritional ones, affecting outcomes, and (3) explore AI's role in identifying or reducing these risks. The goal is to enhance personalized, data-driven post-surgical nutritional care to boost recovery and long-term oral health.

## 2 Methodology

### 2.1 Study design and protocol

**Figure 1:**
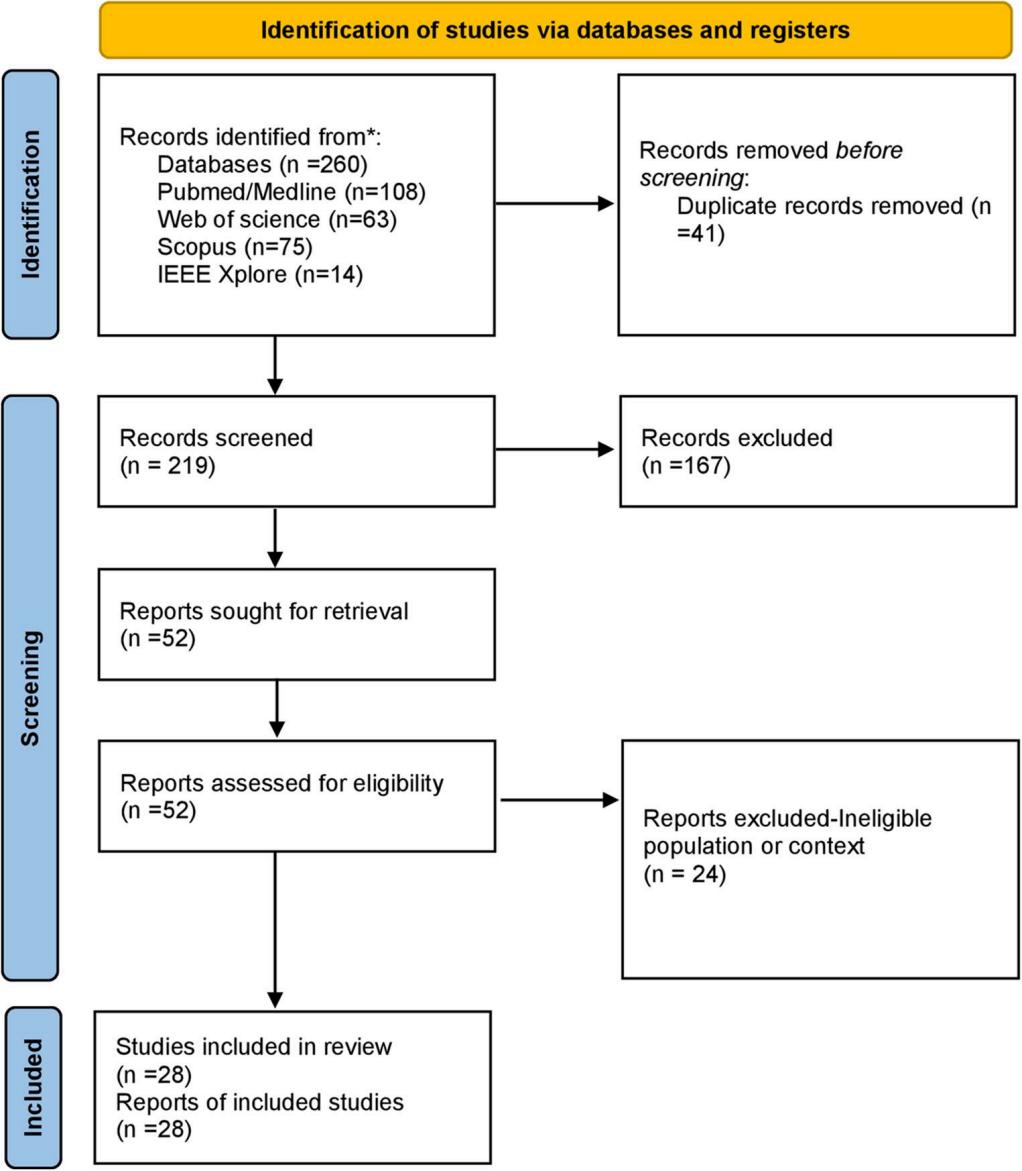
PRISMA guidelines-based workflow.

We conducted a scoping review following established guidelines (PRISMA-ScR) to broadly map the literature on AI applications and nutritional risk factors in post-periodontal surgical care. A scoping review approach was chosen due to the topic's interdisciplinary nature and the expectation of heterogeneous evidence (ranging from clinical studies to technology-focused papers). The review protocol was defined *a priori*, including objectives, eligibility criteria, and methods for evidence charting. We framed our research question using the PCC (Population-Concept-Context) framework as follows: *Population*, patients who have undergone periodontal surgical procedures; Concept, the role of artificial intelligence in nutrition care management and associated risk factors; Context, postoperative periodontal healing and dietary/nutritional management (Figure 1).

### 2.2 Eligibility criteria

We included peer-reviewed articles published in English between January 2019 and October 2025 that met the following criteria: (a) studies focusing on artificial intelligence applications in dentistry (especially periodontology or oral surgery) with relevance to patient management or risk assessment; (b) studies focusing on nutritional care, diet, or metabolic risk factors in the context of periodontal disease or oral surgical recovery; or (c) studies explicitly examining risk factors for healing outcomes after periodontal surgeries, including nutritional aspects. All study designs were eligible (e.g., randomized trials, cohort studies, cross-sectional studies, case series, reviews, and conference papers) as long as they provided insight into either AI tools in periodontal care or the impact of nutrition-related factors on periodontal surgery outcomes. We excluded non-peer-reviewed literature (news articles, websites, and preprints that had not undergone peer review) to ensure reliability. We also excluded papers focused solely on general dentistry AI with no relevance to periodontics, as well as those on nutrition in oral health that did not address periodontal surgery or disease.

### 2.3 Search strategy

A comprehensive literature search was performed in September 2025 using multiple databases: PubMed/MEDLINE, Web of Science, Scopus, and IEEE Xplore (to capture relevant computing literature). The search combined keywords and controlled terms across three domains: *Periodontal Surgery*, *Nutrition*, and *Artificial Intelligence*. Example search strings included: “periodontal surgery OR periodontitis AND nutrition OR diet OR malnutrition AND artificial intelligence OR machine learning OR predictive analytics”, and “periodontal AND (diet OR nutrition OR healing) AND (machine learning OR expert system OR decision support)”. We also searched specific journals (e.g., Journal of Clinical Periodontology, Clinical Nutrition, Frontiers in Oral Health, Journal of Dental Research). We scanned the reference lists of relevant articles for any additional studies.

**Figure 2:**
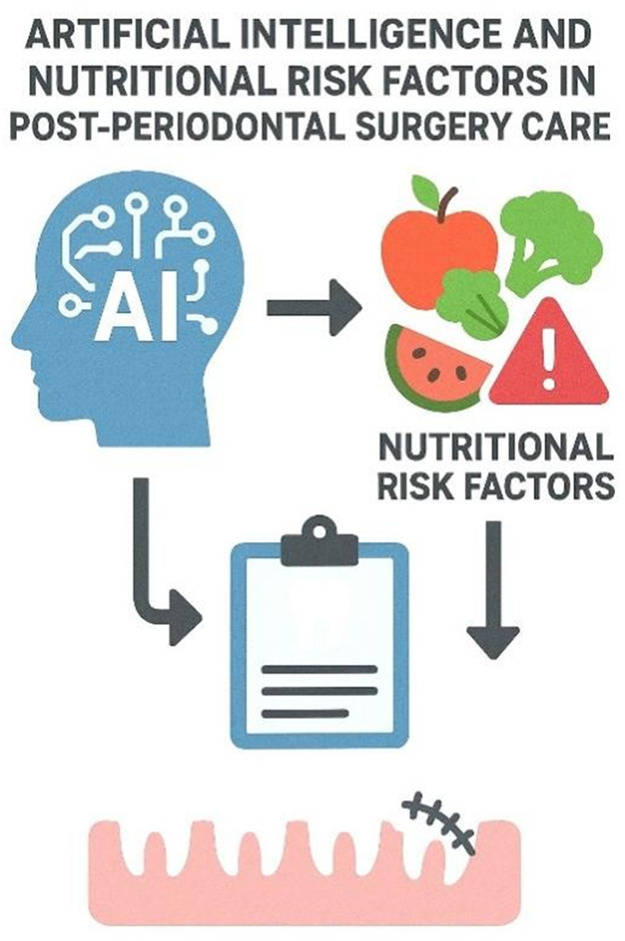
Nutritional risk factors in periodontal surgical care.

All search results were imported into a reference manager, and duplicates were removed. Two reviewers independently screened titles and abstracts for potential relevance. Any discrepancies were resolved through discussion, with a low threshold for including articles for full-text review, thereby maximizing the capture of pertinent literature. Full texts of 52 articles were retrieved and assessed against the inclusion criteria. Ultimately, 28 publications were included in the review (see Figure 2 for a flow diagram of the study selection process). The included articles comprised 15 research studies (5 focused on AI in periodontics, four on nutrition and periodontitis, and six on risk factors/outcomes of periodontal therapy) and 13 review or commentary papers (Figure 1).

### 2.4 Data charting and synthesis

A data-charting form was developed to extract key information from each included source. For each study or article, we recorded the following details: citation information (author, year, journal), study design/type, objective, AI method or nutritional aspect investigated, main findings related to AI or risk factors, and any explicit mention of implications for nutrition management post-surgery. Given the broad scope, we categorized the literature into thematic groups for synthesis: (1) AI in Periodontal Care—covering diagnosis, prognosis, and decision support tools. (2) Nutritional Factors in Periodontics—covering diet, supplements, and metabolism in periodontal disease or healing. (3) Risk Factors for Postoperative Outcomes—clinical or behavioral factors affecting healing after periodontal interventions. (4) Intersections of AI and Nutrition—any studies or concepts directly combining these.

We summarized evidence qualitatively, using counts and proportions to describe publication types and risk factors. No formal quality assessment was conducted due to the scoping nature, but study designs and sizes were noted to contextualize the strength of the evidence. Two reviewers synthesized results collaboratively, reporting findings narratively, supported by summary tables and figures for clarity.

## 3 Results

### 3.1 Characteristics of the included studies

The 28 included publications spanned a diverse range of study designs and focal topics. Table 1 summarizes representative studies and illustrates the convergence of AI, periodontal treatment, and nutrition-related factors. The majority of included papers (*n* = 18) were published in 2020 or later, reflecting a recent surge in interest at the intersection of AI and dentistry. Geographically, research originated in multiple regions (North America, Europe, and Asia), underscoring the topic's global relevance. About one-third of the papers were review articles (including narrative reviews and scoping reviews) that synthesize developments in AI for periodontology or discuss the role of nutrition in oral health. The remaining two-thirds consisted of original research, which included clinical studies examining the outcomes of periodontal therapy (with analyses of risk factors such as nutritional status) and data science studies that developed or validated AI models for dental applications. Notably, although no single study explicitly combined AI and nutritional management in periodontal patients, complementary evidence emerged from separate lines of inquiry—some focused *n* AI-based risk prediction in periodontics, others on nutritional risk factors in periodontal healing. We synthesized these parallel strands to address the questions raised in our review.

**Table 1:** Examples of recent studies at the interface of AI, periodontal care, and nutrition.

Study (Year)	Design/Type	AI Technique/Focus	Key Findings (Relevance)
Walter et al. (2025) (15)	Retrospective cohort (multi-center)	Machine learning predictive model (gradient boosting, etc.) to predict periodontal pocket reduction after therapy (Step II and surgical)	The ML model predicted post-therapy outcomes, identifying baseline PPD and tooth-level factors as the most important predictors of healing. The use of systemic antibiotics also influenced outcomes. Demonstrates AI's ability to highlight key risk factors for poor response, which could inform personalized postoperative care
Gu et al. (2025) (16)	Retrospective clinical study (121 patients)	No AI; logistic regression risk analysis for complications after periodontal flap surgery	Postoperative complications (pain, infection, delayed healing) occurred in ∼45% patients. Age (older age +12% risk per year) and smoking were significant risk factors, along with greater preoperative attachment loss (disease severity). Emphasizes the need for tailored management (possibly nutritional support) in older, high-risk patients
Sarakbi et al. (2025) (17)	Scoping Review (30 studies)	AI in periodontal maintenance (diagnosis, risk assessment, etc.)	AI (especially deep learning on radiographs) achieved >90% accuracy in periodontal disease detection. Predictive analytics, utilizing combined data (clinical and demographic), enabled personalized risk assessments and treatment plans, aligning with precision medicine. Highlights AI's role in risk stratification, which could be extended to nutritional risk profiling
Lettieri et al. (2025) (18)	Review/Commentary (geriatric focus)	Not applicable (review)	Identified impaired masticatory function as a determinant of malnutrition in older people. Older adults with poor chewing had significantly lower intake of protein, fiber, and vitamins (A, C, etc.) and a higher risk of malnutrition, even after adjusting for confounders. Recommends integrating dental care with nutritional support, which is particularly relevant for post-surgery patients experiencing mastication issues
Khan et al. (2024) (19)	Narrative Review	Overview of AI in periodontology and implantology	Summarized AI successes in periodontics: e.g., automated bone loss measurement, periodontal risk assessment (PRA) tools, prediction of treatment outcomes. Concluded AI can serve as a decision aid to improve clinician effectiveness. Stressed need for regulatory approval and training for clinical adoption. (Sets stage for using AI in clinical decision support, including nutrition-related decisions)
Li et al. (2025) (20)	Cross-sectional (NHANES analysis)	Statistical analysis (no AI)	The study revealed that socioeconomic factors are strongly associated with the prevalence of periodontitis. Periodontitis shares risk factors with chronic diseases, including smoking, poor diet, and socioeconomic challenges. Implies that patients from lower SES backgrounds might need additional support (nutritional counseling, etc.) after surgery
Sharma et al. (2020) (21)	Review/Case study (health system data)	EHR-based malnutrition screening with ML decision support	Highlighted a pipeline where machine learning algorithms integrated with EHR provide decision support to identify and manage patients at high risk of malnutrition. While not dental-specific, it demonstrates AI's capability for nutritional risk stratification in clinical practice—the concept could be applied to flag periodontal patients who need nutritional interventions

### 3.2 AI applications in postoperative care for periodontal patients

Several included studies and reviews describe AI applications in periodontology that, although not explicitly focused on nutrition, lay the groundwork for improved post-surgical patient management. The use of machine learning (ML) to predict periodontal treatment outcomes is particularly relevant. Walter et al. developed an ML model to predict changes in periodontal probing depth after non-surgical and surgical therapy. The model could distinguish sites that would improve (pocket reduction) from those that would not, thereby identifying “non-responding” sites or patients. Importantly, the algorithm's analysis revealed which features were most predictive: initial pocket depth was the top predictor, and others, such as tooth type and whether the patient received adjunctive antibiotics, also influenced the outcome (15). This illustrates AI's ability to process complex clinical data and surface key risk factors. In practice, such an AI tool could be used pre- or post-surgery to flag sites at risk of poor healing. For example, a deep pocket on a specific tooth in a patient without antibiotic coverage might be predicted not to heal fully, alerting the clinician to intensify localized care or arrange follow-up. Although nutrition was not a variable in that study, one could imagine including nutritional indicators (e.g., serum albumin, BMI, dietary quality scores) into similar models in the future to enhance their prognostic power for healing outcomes (16).

Artificial intelligence in periodontal care falls into categories based on function and technology. Diagnostic uses deep learning, especially convolutional neural networks, to detect bone loss and disease patterns from radiographs and images. Prognostic models use machine learning algorithms like gradient boosting, random forests, and neural networks to predict treatment response, healing, and disease progression from clinical data. Decision-support systems combine rule-based expert systems and hybrid AI to incorporate patient risk factors, clinical guidelines, and analytics for personalized treatment and postoperative care (15). Most existing AI-based periodontal models mainly use clinical severity markers (like pocket depth and attachment loss), treatment variables, and demographics. Nutritional biomarkers such as BMI, serum albumin, vitamin D, and micronutrients are seldom included despite their importance for wound healing. Broader medical AI research shows low BMI and hypoalbuminemia predict delayed healing and complications, implying that adding nutritional biomarkers could improve periodontal prediction models' accuracy and personalization (16).

Another domain is clinical decision support systems (CDSS) for periodontal care. The European Federation of Periodontology's 2025 Digital Innovation Award went to “PerioPredict,” an AI-assisted application for periodontal decision-making. PerioPredict allows clinicians to input structured clinical data (probing depths, radiographic findings, etc.) and receive real-time insights into disease classification and likely treatment outcomes. While the AI is “assistant” level (requiring manual input and clinician oversight) rather than fully autonomous, it demonstrates how AI can enhance consistency and foresight in periodontal treatment planning. For instance, the system can predict the probability of success for procedures such as gingival recession coverage based on specific patient factors. This kind of AI-driven tool could easily be extended to incorporate nutritional risk by including a module that analyzes patient diet or lab data and warns if poor nutritional status might jeopardize surgical success. Many AI-driven health platforms are trending toward a holistic approach, integrating various data streams. Sarakbi et al. noted that AI in periodontal maintenance enables “highly personalized care strategies” by integrating diverse datasets—including clinical measures, demographics, and lifestyle factors—to create tailored risk profiles (17) (Figure 2). Lifestyle factors implicitly include diet, so a smart AI could correlate nutritional deficiencies with slower periodontal recovery and factor that into its risk calculations. Expert systems, a classical form of AI, have also been explored in dentistry. While older in concept, they use a knowledge base and inference rules to provide recommendations. For example, an expert system for postoperative nutrition might encode guidelines: if a patient has diabetes, suggest a low-sugar, high-protein diet; if they have had a bone graft, recommend calcium and vitamin D; if they are elderly with chewing difficulties, suggest high-calorie, soft foods or supplements. Though not specifically documented in periodontics, this aligns with AI-based clinical pathways. In general surgery, predictive analytics forecast nutrition-related complications, such as the need for feeding support after gastrointestinal surgery, demonstrating that AI trained on perioperative data can predict malnutrition or intervention needs with reasonable accuracy (18).

Image recognition can detect what food characteristics and make an educated prediction about how healthy it is (19, 20). People with periodontal disease might use this kind of software to keep track of how much protein they eat or to make sure they get enough vitamins as they heal. New AI chatbots and virtual nutritionists can provide people personalized advise and motivation about what to eat. They can give clients individualized advise that is quite similar to what a genuine dietitian would give, which helps them follow the rules. For example, an AI chatbot might tell patients to drink enough water or eat certain foods every day. It might also answer questions like, “Can I eat X after my gum surgery?” This fills the gap between seeing the doctor and taking care of yourself during the essential 1–2-week recovery time, as proven by algorithms (17, 19, 20).

### 3.3 Risk factors influencing nutritional care post-periodontal surgery

We observed a consistent set of risk variables in the literature we read that affect either the healing process following periodontal surgery or the nutritional state of patients in this situation. Table 2 displays these significant components, their impacts, and the data derived from the examined sources. A lot of risk factors are linked to each other or arise at the same time in some groups of patients. For instance, elderly people could have more than one health issue and be in a worse financial situation. There are four main groups of risk factors: patient demographics (age, sex), systemic health factors (comorbid conditions, immune status), lifestyle/behavior (smoking, diet quality, oral hygiene compliance, alcohol consumption), and psychosocial factors (education, income, social support).

**Table 2:** Major risk factors affecting nutritional management and healing after periodontal surgery.

Risk Factor	Influence on Nutrition/Healing Post-Surgery	Supporting Evidence (2019–2025 literature)
Advanced Age	Older patients have a higher malnutrition risk and slower wound healing. Often present with oral frailty (tooth loss, reduced chewing efficiency) leading to reduced dietary intake. Healing capacity and immune response decline with ag	Each additional year of age ↑ increases the complication risk by 12% after periodontal surgery. Elderly with poor mastication (18) (Li et al.) are low socioeconomic older adults at risk of periodontitis, partly due to disparities in health and nutrition issues
Comorbidities (e.g., Diabetes)	Systemic diseases like diabetes mellitus impair healing (due to microvascular changes and immune dysfunction) and often require dietary modifications. Malnutrition or specific deficiencies may coexist with illness. E.g., people with diabetes need blood sugar control (affecting diet) and have a higher infection risk. Other conditions (cardiovascular, renal, etc.) can also affect nutritional needs and dietary tolerance	Prior studies cited diabetes as a risk factor for adverse outcomes in periodontal surgery. Diabetes and obesity are known periodontal risk factors that also impact diet and inflammation (16). Undernutrition exacerbates chronic conditions; identifying patients with comorbidities at risk enables the delivery of tailored care
Poor Preoperative Nutritional Status (malnutrition, low BMI, or micronutrient deficiencies)	Patients who are undernourished or deficient in key nutrients before surgery have less reserve to support tissue repair. Protein-energy malnutrition impairs collagen synthesis and immune function; low levels of vitamins (C, D, and B complex) or minerals (zinc) can delay the healing of gums and bones. Such patients may experience prolonged recovery and are more prone to infection	Vitamin D-sufficient patients had better periodontal surgical outcomes (more bone gain) than deficient patients.This highlights the importance of identifying malnourished patients through EHR/AI and intervening to improve outcomes
Dietary Compliance (Post-surgery diet adherence)	Low compliance with recommended dietary guidelines (e.g., not maintaining a soft diet when advised, or not consuming adequate protein/vitamins) can lead to mechanical disruption of the surgical site or insufficient nutrient supply for healing. Compliance issues may stem from a lack of understanding, cultural food preferences, or practical difficulties in meal preparation	There is a stressed need for *individualized diet planning* and assessing food preferences to improve compliance. Notes that if diets are too complicated or unpalatable, patients will not adhere. Identified patient compliance as “the most important factor” for outcome—patients must be educated and motivated. In periodontal maintenance, poor compliance (with oral hygiene or diet) undermines the benefits of any therapy
Impaired Oral Function (chewing, swallowing ability)	Difficulty chewing due to missing teeth, pain, or ill-fitting dentures leads to avoidance of hard or fibrous foods, reducing overall nutrient intake (particularly protein, fruits, and vegetables). This can result in a softer, often carbohydrate-heavy diet that may be poorer in protein and micronutrients. In extreme cases, patients may eat too little, losing weight and muscle (sarcopenia). Poor oral function thus directly contributes to malnutrition and can form a vicious cycle (malnutrition further reduces muscle strength, etc.)	Defines “oral frailty”—decline in oral function linked to disability and malnutrition; poor chewing is a determinant of malnutrition in older adults. Recommends oral rehabilitation and nutritional support in tandem to break the vicious circle
Smoking and Alcohol Use	Tobacco smoking is a well-documented risk factor for poor periodontal healing and also has nutritional implications (smokers often have lower vitamin C levels and appetite suppression). Smoking causes vasoconstriction and impairs immune function in oral tissues. Excessive alcohol can interfere with nutrition (leading to deficiencies like B-vitamins) and wound healing, and may also reduce compliance (intoxication or neglecting diet)	Smoking significantly increased the risk of complications post-surgery. Lists smoking and alcohol as lifestyle risk factors that can exacerbate periodontitis and systemic inflammation. These factors indirectly affect nutrition by altering metabolism and patient behavior. Low socioeconomic status often correlates with higher smoking rates; smoking + poor diet combo drives worse outcomes
Socioeconomic Status (SES) (low income, education)	Lower SES is linked to limited access to nutritious foods (food insecurity), less health literacy regarding diet, and fewer resources to afford supplements or special diets. Such patients may rely on cheap, calorie-dense but nutrient-poor foods. They also might have less access to supportive services (dietitians, follow-up care). Low SES is thus associated with both a higher prevalence of periodontal disease and a worse nutritional status, creating a double jeopardy for healing	Found that low income and related SDoH are significantly associated with periodontitis prevalence. Mentions “limited access to healthy food” as part of the causal pathway. Identifies low SES as a patient risk factor for periodontitis and peri-implantitis, alongside diet and smoking (stating these often cluster). Suggests assessing patients’ ability to procure and prepare recommended foods; encourages establishing a support network for food preparation if needed

As seen in Table 2, age and overall health, especially diabetes, are two of the most common causes. Lettieri et al. say that older patients not only take longer to heal, but they also have a number of problems, such as oral fragility, that could leave them malnourished. Numerous authors emphasized the necessity for comprehensive geriatric care encompassing dental and dietary services. For instance, many elderly patients who were malnourished also had poor oral health. This led to the idea that nutritionists should work with dentists on a regular basis. This indicates how closely results are tied to age and nutrition (18).

Identified nutritional risk factors affect periodontal healing through biological and behavioral mechanisms. Age and diabetes impair microvascular perfusion, collagen synthesis, and immune function, delaying wound repair. Reduced oral function limits intake of proteins, vitamins, and micronutrients, weakening tissue regeneration. Low socioeconomic status restricts access to nutrient-rich foods and supplements, raising malnutrition risk. Poor nutritional status and low dietary compliance decrease substrates for fibroblast growth, angiogenesis, and epithelial migration, increasing chances of delayed healing, wound dehiscence, and infections.

**Figure 3:**
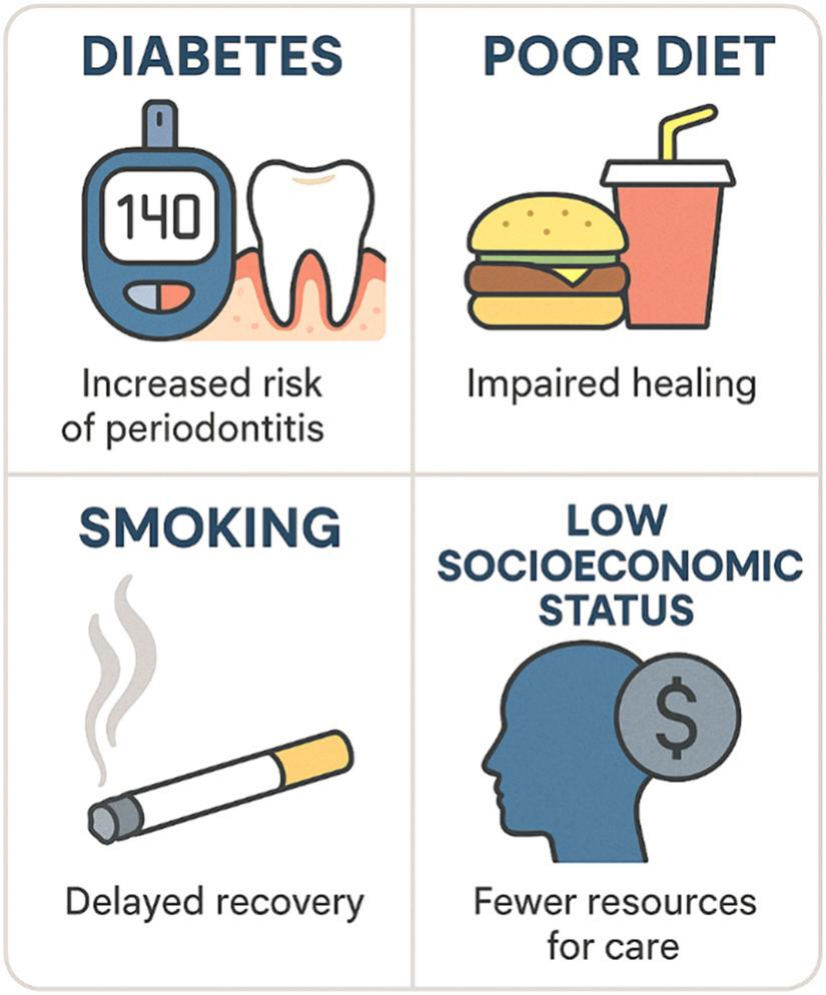
Risk factors associated with healing outcomes.

Diabetes is a major risk factor for gum disease because it makes it harder for wounds to heal and makes it harder to get the nutrients you need. Individuals with diabetes must vigilantly monitor their blood glucose levels. High blood sugar levels weaken the immune system and collagen synthesis, which makes it more likely that infections and illnesses will return back (Figure 3). None of the AI models we examined explicitly incorporated diabetes, yet it seems probable that it is crucial. Diabetes is an important part of standard risk assessments, including the Periodontal Risk Assessment tool. It's likely that AI models in the future will do the same thing. One of the studies identifies age, BMI, blood pressure, and smoking as risk factors, with BMI functioning as a partial indicator of nutritional status, suggesting that metabolic metrics must be integrated into AI risk models (19).

Lifestyle factors, such as smoking and diet quality, frequently coexist and collectively impact outcomes. Smoking has always been linked to slower healing and other problems. It reminds us that after surgery, a patient should seek nutritional counsel that includes instructions on how to stop smoking to help them heal as quickly as possible. Lastly, socioeconomic class has a big but less obvious effect. It affects practically everything: what foods the patient can buy, if they have a caretaker or family member who can assist them cook soft meals, how well they follow orders, and their other bad habits that could hurt them. An AI model could change its risk predictions based on socioeconomic status-(SES) proxies such as education level, insurance status, and local deprivation index. Li et al. clearly show that bad socioeconomic situations are linked to higher rates of periodontitis. This means that certain people may need additional help after the surgery. In light of our review, we interpret this to indicate that clinicians should expect that a low-SES patient may require not only standard postoperative instructions but also additional resources—such as supplying nutritional supplement samples, organizing follow-up calls to reinforce dietary guidance, or facilitating connections with community meal services during recovery (19, 20). AI could help by figuring out what people need. For instance, if a low-income patient is undergoing significant periodontal surgery, the hospital's electronic health record (EHR) might instantly set up a consultation with a nutritionist, just way it does for an orthopedic operation in a malnourished patient.

### 3.4 Integrating AI to address nutritional risk factors

AI and risk variables work together to achieve tremendous progress. AI could be able to aid with nutritional problems, even though there isn't much strong proof. In periodontology, AI, like predictive analytics, can use factors like age, smoking status, indices, BMI, albumin levels, and eating habits to guess how a disease will get worse and how long it will take to get better. There aren't many direct studies, but related research uses regular ICU data to guess who is malnourished. This means that similar technologies may be used to discover people who are at risk after surgery, which would make it easier to respond swiftly. AI can also keep track of what you consume via wearables and apps. It can utilize smart counters or image logs to keep track of how you eat and how healthy your meals are. This gives you the ability to manage your diet in a way that works for you. The study focuses on precision nutrition, which implies making meals that are specific to each person's genes, microbiome, and way of life. This is something AI can help with. For instance, looking at the oral microbiota along with the diet may help figure out if probiotics or prebiotics are needed to improve dental health. This fits with the assumption that chewing has an effect on microbiota and health, especially in elderly persons. AI employs a lot of different types of data to make individualized care plans that include changing the microbiome, diet, exercise, and prostheses (21).

## 4 Discussion

This scoping research aimed to examine the amalgamation of artificial intelligence with nutritional therapy in post-periodontal surgery patients and to discern the risk factors influencing patient outcomes. The findings suggest the emergence of a nascent discipline—direct research on AI for nutrition in dentistry remains constrained—yet the fundamental components for progress are present. Combining how AI may help manage periodontal disease, largely focusing on diagnostics and risk prediction and how food and other systemic factors can affect periodontal healing. We can identify effects, problems, and future paths for using AI in nutritional management for people who are having periodontal surgery.

### 4.1 Implications for clinical practice

The acknowledged risk factors underscore the imperative for periodontists and dental surgeons to adopt a comprehensive, patient-centered approach in postoperative care. When clinicians see signs like age, diabetes, and poor nutrition at the beginning, they should be particularly careful. Our research shows that, if they are used, these “extra” steps depend on the clinician's experience and gut feeling. AI can help make this process more reliable and better. Not every dental surgeon, for instance, may be good at evaluating diet. An AI program could help keep patients safe by periodically checking them against objective standards and proposing referrals or actions when certain thresholds are reached. In real life, dental offices might include a short nutrition screening questionnaire to the electronic records of patients who need surgery. An AI machine can quickly sort through these kinds of responses and health information to classify patients into groups based on how at risk they are for nutrition problems. Patients who are at high risk could automatically get a more interdisciplinary treatment plan, like working with a dietitian and having follow-ups more often. This could lead to better outcomes by dealing with issues before they happen, rather than waiting for them to happen (such a wound infection or weight loss in the patient) (22).

Another important benefit is that it will help patients learn more and get more involved. Patients frequently fail to comprehend the significance of nutrition in the healing process. As we spoke about before, chatbots and other AI-powered apps could make learning more fun and engaging than books. For instance, a chatbot might ask the patient, “Did you eat something with protein in it for lunch today?” and then give them some choices. It can also answer patient questions at any time of day or night. Chatbots can help people stick to changes they make to their lives, according to other studies. People with periodontal disease need to eat well and keep their teeth clean for a few weeks after surgery. An AI assistant can support the healthcare workers by giving them daily motivation and advice. This could lead to higher compliance and, as a result, better results. People who don't know much about health might find these kinds of technologies quite helpful. The AI can help people understand and connect with each other better, maybe even in the patient's own language, by filling in gaps in knowledge and SES (23).

### 4.2 Benefits and efficacy

AI can assist lower the risk of infections, wound problems, and poor tissue regeneration by finding patients who are at high risk and giving them nutritional support. Eating well may also help with pain and energy levels, which can help heal faster. AI helps allocate resources by identifying individuals who need specific therapy and reducing superfluous visits. Data quality and availability are still issues, even when everything seem fine. AI needs good data, but it's hard to make progress in medicine because the data is housed in different places and is not the same in each one. Our research indicates that dental offices infrequently gather nutritional data, and there is a scarcity of studies examining dietary or blood nutrient levels. Subsequent study ought to integrate these factors and publish statistics, fostering collaboration between dental and nutrition professionals to enhance data techniques, including the incorporation of mini-nutritional assessments in surgical trials. Numerous AI models utilized in dentistry have yet to be validated in real clinical settings. They can't be utilized in real life even though they say they are very accurate (>90%) because of rules, how they fit into the workflow, and how doctors are trained. Ethical considerations, including as patient privacy, bias, and human oversight, are very important. Healthcare personnel employing AI in patient care must comprehend and elucidate AI recommendations (“explainable AI”) to facilitate appropriate patient disclourse.

### 4.3 Limitations

AI-based nutritional risk assessment systems must address fairness and privacy. Mitigate socioeconomic bias by using balanced data, explicitly include social determinants, and audit model outputs across demographics. Use explainable AI for transparency, helping clinicians spot bias. Ensure privacy with anonymization, encryption, access controls, and compliance with data standards. These measures support ethical, transparent, and equitable AI-driven nutritional care. There were limitations in our study. We combined data from several study designs and looked at more than just the immediate results. For example, we made predictions on how well AI will be able to handle nutritional data. Currently, there is no model that accomplishes this within the field of periodontics. These are not facts; they are guesses. We may have missed any recent studies or research that weren't in English, but we tried to be as thorough as possible by looking at a wide range of topics across six years. There is insufficient direct research about the application of AI in food management for periodontal patients, highlighting a research deficiency. Future research may evaluate AI therapies in comparison to traditional treatments or investigate nutritional factors affecting recovery, such as vitamin D levels or blood albumin concentrations. People from diverse areas need to work together. This is shown by the fact that patient registries maintain track of dental and dietary information to assist make AI models. Clinicians will be more likely to trust and look at model predictions if they use explainable AI. Dental schools need to modify their curriculum pertaining to nutrition.

Current AI-powered nutritional advisors lack cultural sensitivity, relying mainly on generic databases. Future systems should include region-specific foods, validated dietary patterns, religious restrictions, and patient preferences as inputs. Training on diverse datasets and applying cultural filters can help AI create personalized, culturally acceptable nutrition plans for periodontal patients worldwide.

## 5 Conclusion

This study examines the influence of AI on the management of dietary issues following periodontal surgery. AI, which includes machine learning and decision support systems, uses a lot of various types of data, such as clinical and lifestyle data, to help doctors make better diagnoses, more consistent risk assessments, and more personalized therapies. AI models can make educated guesses about how well someone will heal depending on their age, the severity of their sickness, and the treatment they are getting, all of which have to do with nutrition. Age, diabetes, not getting enough food, bad eating habits, problems swallowing, and low income are all nutritional risk factors that could impede down healing after surgery. Nutrition, education, and monitoring are some of the things that can help lower a number of dangers. At the moment, periodontal care depends on the clinician's judgment for controlling nutrition. AI, on the other hand, offers a systematic, evidence-based way to do this. AI can aid with nutrition care and rehabilitation after surgery, but for this to work, dental doctors, nutritionists, and data scientists need to work together and have access to good data. The literature shows that AI is moving swiftly from 2019 to 2025, and there will likely be experimental programs in the future. In the end, a patient-centered, AI-driven approach may be able to tailor nutritional support to each person, speeding up recovery and making dental health better. It could also make periodontal therapy better by making it more accurate and thorough

## Data Availability

The original contributions presented in the study are included in the article/Supplementary Material, further inquiries can be directed to the corresponding author.
